# Intake of citrus fruits and vegetables and the intensity of defecation urgency syndrome among gynecological cancer survivors

**DOI:** 10.1371/journal.pone.0208115

**Published:** 2019-01-02

**Authors:** Maria Hedelin, Viktor Skokic, Ulrica Wilderäng, Rebecca Ahlin, Cecilia Bull, Fei Sjöberg, Gail Dunberger, Karin Bergmark, Andrea Stringer, Gunnar Steineck

**Affiliations:** 1 Regional Cancer Center West, Sahlgrenska University Hospital, Gothenburg, Sweden; 2 Department of Oncology, Division of Clinical Cancer Epidemiology, Institute of Clinical Sciences, Sahlgrenska Academy at the University of Gothenburg, Gothenburg, Sweden; 3 Department of Health Care Sciences, Ersta Sköndal Bräcke University College, Stockholm, Sweden; 4 School of Pharmacy and Medical Sciences, Division of Health Sciences, University of South Australia, Adelaide, Australia; 5 Department of Oncology and Pathology Division of Clinical Cancer Epidemiology, Karolinska Institutet, Stockholm, Sweden; University Hospital Llandough, UNITED KINGDOM

## Abstract

**Background:**

Despite the experimental evidence that certain dietary compounds lower the risk of radiation-induced damage to the intestine, clinical data are missing and dietary advice to irradiated patients is not evidence-based.

**Materials and methods:**

We have previously identified 28 intestinal health-related symptoms among 623 gynaecological-cancer survivors (three to fifteen years after radiotherapy) and 344 matched population-based controls. The 28 symptoms were grouped into five radiation-induced survivorship syndromes: *defecation-urgency syndrome*, *fecal-leakage syndrome*, *excessive mucus discharge*, *excessive gas discharge* and *blood discharg*e. The grouping was based on factor scores produced by Exploratory Factor Analysis in combination with the Variable Cutoff Method. Frequency of food intake was measured by a questionnaire. We evaluated the relationship between dietary intake and the intensity of the five syndromes.

**Results:**

With the exception of excessive mucus discharge, the intensity of all syndromes declined with increasing intake of citrus fruits. The intensity of defecation-urgency and fecal-leakage syndrome declined with combined intake of vegetables and citrus fruits. The intensity of excessive mucus discharge was increased with increasing intake of gluten.

**Conclusion:**

In this observational study, we found an association between a high intake of citrus fruits and vegetables and a lower intensity of the studied radiation-induced cancer survivorship syndromes. Our data suggest it may be worthwhile to continue to search for a role of the diet before, during and after radiotherapy to help the cancer survivor restore her or his intestinal health after irradiation.

## Introduction

Radiotherapy is a common treatment for cancer in the pelvis and lower abdomen. Between 70% and 90% of patients receiving radiotherapy treatment have been reported to develop acute gastrointestinal symptoms [1, 2], and for many patients the treatment will induce lifelong survivorship diseases [3, 4]. An underlying cause is fibrosis, the end result of radiation-induced degeneration of the crypts in the gut wall, damage to the endothelium and inflammatory processes [5]. Possibly, the colonic wall attempts to repair itself by crypt fission [6], but insufficiently to prevent fibrosis.

Even though it is known that radiation induces intestinal fibrosis and that survivors suffer, few preventive or curative interventions have been successfully used in clinical practice [7–9]. A diet high in fruit and vegetables maintains a healthy function of the normal colon and the protective mucus layers are reduced by a diet without fiber [10]. There is no body of scientific evidence available today that shows that patients should avoid dietary fiber [7], but radiated patients are nevertheless often given the advice to do just that, avoid dietary fiber intake.

Fruits and vegetables contain non-starch polysaccharides such as pectin. Pectin is a highly complex polysaccharide fiber with soluble and viscous properties (so-called soluble fiber) and has been found to have chemo-preventive and radio-protective properties [11]. For example, pectin has been suggested to increase intestinal crypt stem cell survival following radiation injury [12] as well as reducing radiation-induced intestinal fibrosis [13]. Pectin also appears to prevent loss of microbial diversity caused by radiation [13].

Soluble fiber is fermented by colonic bacteria into short-chain fatty acids including butyrate [14] that have an anti-diarrheal, anti-inflammatory, anti-oxidative, anti-carcinogenic and anti-fibrogenic effects [13, 15, 16]. Butyrate acts as an energy source for the colonic epithelial cells, providing up to 70% of their energy requirements [17]. A diet low in fiber leads to starvation of the fiber-degrading gut microbiota. This promotes the expansion of bacteria specialized in degrading the host-secreted mucus glycoproteins. This in turn leads to erosion of the protective mucus barrier lining the gut wall [10]. Furthermore, our own preclinical studies indicate that a diet combining non-fermentable fiber with a larger volume of fermentable fiber is protective against radiation-induced gut wall damage (unpublished data). This protective effect probably results because the soluble fiber is transported with the insoluble fiber distally into the large intestine and rectum, where it is fermented and free fatty acids are produced [18].

We have previously documented patient-reported long-term symptoms one by one, using questionnaires specially developed for this approach to produce what we call atomized clinimetric symptom documentation. Self-reported information on long-term symptoms was collected from a large cohort of cancer survivors treated with radiation to the pelvic area and from matched control subjects [19].

Using a new approach, the documentation of intestinal related symptoms was analyzed using factor analysis [3] making it possible for us to identify five survivorship syndromes: defecation-urgency syndrome, leakage syndrome, excessive mucus discharge, excessive gas discharge, and blood discharge. These syndromes thus appear to underlie the nearly 30 intestinal-related symptoms reported by cancer survivors with a history of radiation-treatment focused on the pelvic area. In the population-based cohort study presented here, we examined the relationship between dietary intake of fruit and vegetables and intensity of the five radiation-induced survivorship syndromes three to fifteen years after treatment with radiotherapy.

## Material and methods

### Study population

The study design and assessment of patient-reported long-term symptoms have been described in detail elsewhere [19]. In brief; between 1991 and 2003, 1800 women were treated with external pelvic radiotherapy for a gynecological malignancy at two clinics in Sweden. The patients had received long-course external-beam radiation therapy, sometimes combined with brachytherapy, either as radical treatment or in addition to surgery [20]. This corresponded by and large to all the relevant gynecological cancer patients in two geographical regions. We excluded survivors born 1927 or earlier, as well as those who were illiterate in Swedish.

Based on the clinical epidemiological tradition for atomized patient-reported outcomes, we constructed a study-specific questionnaire with a section focusing on intestinal health. The atomized questions were based on semi-structured interviews and had wordings as close as possible to those used by the survivors in the transcribed interviews.

An introductory letter was sent to, 823 gynecological-cancer survivors and 478 matched (age, residence) population-based controls, explaining the objectives of our study and emphasizing that participation in the study was voluntary (detailed information is found in Supporting information in reference [3]). One week later, an interviewer phoned each informant. Those giving informed oral consent to participate received a postal questionnaire along with a letter and additional information. To maintain anonymity, each questionnaire contained a number for identification. Six hundred fifty (79%) survivors and 344 (72%) controls returned a questionnaire. An error in the matching procedure led to a younger control population (mean age 58.0) compared to the cancer survivors (mean age 64.4) which was adjusted for in the analyses. No children were included. The study was approved by the Regional Ethical Review Board (2005/1424-31/4), Stockholm, Sweden. The Ethical Review Board received all information and material before they gave their approval.

### Radiation-induced survivorship symptoms

We utilized 28 different atomized symptoms related to intestinal health. For example, concerning toilet dependency we asked: “Have you had sudden defecation urgency that requires immediate toilet visits during the last six months?” Answers were given on a person-incidence scale (“No”, “Yes, occasionally”, “Yes, at least once a month”, “Yes, at least once a week”, “Yes, at least three times a week”, and “Yes, at least once a day”). Survey questions are presented in S1 File, also described in detail elsewhere [3, 19]. Through factor scores, five radiation-induced survivorship syndromes were identified (see statistical part below) and are described in detail elsewhere [3].

### Dietary intake

The participants were asked to evaluate how often during the past six months they had eaten citrus fruits, beans and lentils, cabbage and broccoli, onion and garlic, grated vegetables, foods with gluten (the four cereals), chocolate, dairy products, spicy food or fatty food. The options given were “daily”, “weekly”, “monthly”, “less than monthly” or “never”. (Note: In Sweden, the term grated vegetables refers to raw carrot or raw beetroot and cabbage).

Total intake of vegetables and citrus fruits was grouped into five categories (at least daily, at least twice a week, at least weekly, 1–3 times a month, less than monthly or never).

The questionnaire included one question about changes in dietary habits; “Have you changed your diet after the completion of radiation therapy?” Those participants who chose the alternative “Not relevant, I have not changed the diet after completion of radiotherapy” were considered as not having changed their dietary behavior after radiotherapy; all other answers were considered as indicating that dietary behavior had changed after radiotherapy.

### Statistical analysis

The factor scores on which the results presented here are based were produced using Exploratory Factor Analysis (EFA) in combination with a method called the Variable Cutoff Method (VCM). The former is an established dimension reduction method, and the latter aims at identifying spurious so-called factor loadings. Both methods are described in full detail elsewhere [3].

EFA models the covariance matrix of vector of stochastic variables X = (X_1_,…,X_n_) in terms of a matrix L of *factor loadings* and a vector F = (F_1_,…,F_k_), k < n, of unobserved, uncorrelated *factors*. The modelling assumption is: X_i—_μ_i_ = l_i1_F_1_ + … + l_ik_F_k_ +ε_i_, where the ε_i_ are unobserved error terms with mean zero and finite variances. Thus, in order to fit an EFA model to data, the l_ij_ and k have to be estimated. Performing EFA using standardized data is thus equivalent to performing EFA with correlation substituted for covariance. Before any calculations were performed, the data were subjected to two preliminary filtrations: individuals were excluded if they had a bowel stoma (n = 20 survivors, no controls), and if more than 30 percent of the data was missing for the 28 variables under study (n = 7 survivors, no controls). Patients who reported that they had an irritable bowel syndrome (n = 23) were not excluded from the analyses. In the final analyses 623 survivors and 344 controls were included.

In this particular application, the estimated Spearman correlation matrix of the data was used as input to the EFA model, since the data are ordinal. Pairwise deletion of missing values was applied when estimating the correlation matrix. The appropriate number of factors, k, was assessed using both parallel analysis [21] and a bootstrap version of Kaiser’s rule [22]. The two methods agreed on the number of factors. The EFA model was then fit using Maximum Likelihood Estimation [23].

The estimated matrix of factor loadings, L, was then subjected to the VCM. The VCM uses the parametric bootstrap to find factor specific cutoff values on factor loadings, (c_1_,…,c_k_), and sets factor loadings such that |l_ij_|<c_j_ to 0, resulting in a sparse or reduced version L’ of L. Before the calculation of the factor scores the data were imputed using a simple mode value imputation per variable, giving data matrix X’. The factor scores were then calculated using L’ as S = X’L’.

To evaluate the relationship between frequency of dietary intake and syndrome intensities (factor scores) of the five radiation-induced survivorship syndromes defecation-urgency syndrome, fecal-leakage syndrome, excessive gas discharge, excessive mucus discharge and blood discharge, we used Spearmans correlation coefficient. Further, the results were presented graphically as mean syndrome intensities plus-minus the standard error of the mean within certain diet intake frequency levels.

## Results

Demographic data, clinical characteristics, and dietary behaviour of the study participants are presented in Table 1. Most of the cancer survivors were older than 60 years of age, and most had been treated for endometrial (58%) or cervical (23%) cancer. Fifty-two percent of the survivors were treated with external beam radiation plus Brachy therapy. Forty-three percent of the women had eaten citrus fruits daily and 3% were never-eaters. Fewer ate beans and lentils daily, 2% among survivors and 6% among control subjects.

**Table 1:** Demographic and clinical characteristics, and dietary intake in gynecological cancer survivors and controls.

	No. (%)			No. (%)	
	Survivors (N = 623)	Controls (N = 344)		Survivors (N = 623)	Controls (N = 344)
**Age at follow up**^**a**^ **- years**			**Intake of beans / lentils**		
29–49	66 (11)	102 (30)	Daily	15 (2)	20 (6)
50–59	100 (16)	80 (23)	Weekly	136 (22)	90 (26)
60–69	245 (40)	78 (23)	Monthly	118 (19)	84 (24)
70–80	212 (33)	82 (24)	Less than monthly	194 (31)	91 (27)
Not stated	0 (0)	2 (1)	Never	111 (18)	42 (12)
**Education**			Missing	49 (8)	17 (5)
Elementary school	194 (31)	69 (20)	**Intake of cabbage / broccoli**		
Secondary school	238 (38)	146 (42)	Daily	23 (4)	30 (9)
College or university	190 (30)	127 (37)	Weekly	252 (40)	149 (43)
Not stated	1 (0)	2 (1)	Monthly	180 (29)	104 (30)
**Smoking**			Less than monthly	120 (19)	50 (15)
Current smoker	143 (23)	88 (26)	Never	19 (3)	7 (2)
Former smoker	191 (31)	108 (31)	Missing	29 (5)	4 (1)
Never smoker	281 (45)	147 (43)	**Intake of grated vegetables**		
Not stated	8 (1)	1 (0)	Daily	165 (26)	95 (28)
**Body Mass Index (kg/m**^**2**^**)**			Weekly	227 (36)	163 (47)
≤18.5	17 (3)	5 (2)	Monthly	85 (14)	37 (11)
18.5–25	270 (43)	163 (47)	Less than monthly	97 (16)	33 (10)
25–30	199 (32)	116 (34)	Never	19 (3)	8 (2)
≥30	99 (16)	43 (13)	Missing	30 (5)	8 (2)
Not stated	38 (6)	17 (5)	**Intake of onion / garlic**		
**Diagnosis**			Daily	88 (14)	64 (18)
Sarcoma uteri	30 (5)		Weekly	322 (52)	209 (60)
Vulvar cancer	6 (1)		Monthly	92 (15)	40 (12)
Vaginal cancer	14 (2)		Less than monthly	59 (9)	16 (5)
Cervical cancer	146 (23)		Never	25 (4)	6 (2)
Endometrial cancer	363 (58)		Missing	37 (6)	9 (3)
Ovarian cancer	50 (8)		**Intake of gluten**		
Fallopian tube cancer	14 (2)		Daily	268 (43)	176 (51)
Not stated	0 (0)		Weekly	134 (21)	73 (21)
**Treatment modality**			Monthly	48 (8)	24 (7)
Surgery + EBRT^b^	47 (8)		Less than monthly	56 (9)	23 (7)
Surgery + EBRT^b^ + BT^c^	338 (54)		Never	75 (12)	29 (8)
Surgery + EBRT^b^ + Chemo^d^	64 (10)		Missing	42 (7)	19 (6)
Surgery + EBRT^b^ + BT^c^ + Chemo^d^	113 (18)		**Intake of citrus**		
EBRT^b^	2 (0)		Daily	269 (43)	143 (42)
EBRT^b^ + BT^c^	27 (4)		Weekly	222 (36)	145 (42)
EBRT^b^ + Chemo^d^	8 (1)		Monthly	55 (9)	25 (7)
EBRT^b^ + BT^c^ + Chemo^d^	23 (4)		Less than monthly	44 (7)	17 (5)
Not stated	1 (0)		Never	20 (3)	6 (2)
			Missing	13 (2)	8 (2)
**Time since Radiotherapy**					
<3 years	46 (7)				
3–5 years	245 (39)				
6–10 years	226 (36)				
11–15 years	92 (15)				
Missing	14 (2)				

^a^Approximate age at follow up. Calculated as 2006 –year of birth.

EBRT^b^ denotes External Beam Radiation Therapy.

BT^c^ denotes Brachy Therapy.

Chemo^d^ denotes Chemotherapy

We found a statistically significant positive correlation between frequency of citrus fruit intake and intensity of the radiation-induced survivorship syndromes, except for excessive mucus discharge (Tables 2 and 3). For intake of the different vegetable groups, there were statistically significant correlations between frequency of intake and intensity of defecation-urgency syndrome and fecal-leakage syndrome (Tables 2 and 3). Frequency of gluten intake was statistically significantly negative correlated with excessive mucus discharge (Tables 2 and 3). We found no statistically significant correlation between the frequency of intake regarding chocolate, dairy products, spicy food or fatty food, with any of the radiation-induced survivorship syndromes (data not shown).

**Table 2:** Spearman correlation coefficient and corresponding P-values for the relationship between dietary intake and radiation-induced survivorship syndromes among gynecological cancer-survivors and population controls.

DIETARY INTAKE	DEFECATION-URGENCY SYNDROME				FECAL-LEAKAGE SYNDROME			
	CANCER SURVIVORS		POPULATION CONTROLS		CANCER SURVIVORS		POPULATION CONTROLS	
	Corr^a^	P^b^	Corr^a^	P^b^	Corr^a^	P^b^	Corr^a^	P^b^
Beans and lentils	**0.09**	**0.024**	-0.02	0.701	**0.11**	**0.012**	0.03	0.616
Cabbage and broccoli	**0.14**	**<0.001**	-0.01	0.811	**0.11**	**0.007**	-0.02	0.647
Onion and garlic	**0.14**	**<0.001**	0.03	0.6	**0.12**	**0.003**	-0.02	0.745
Grated vegetables	**0.18**	**<0.001**	0.03	0.537	**0.19**	**<0.001**	-0.01	0.895
Gluten	-0.07	0.117	-0.05	0.352	-0.04	0.351	-0.04	0.499
Citrus fruits	**0.16**	**<0.001**	0.07	0.228	**0.14**	**<0.001**	0.06	0.284
Citrus fruits and vegetables	**0.15**	**<0.001**	0.07	0.187	**0.16**	**<0.001**	0.07	0.228

^a^ Spearman correlation coefficient.

^b^ P-value

**Table 3:** Spearman correlation coefficient and corresponding P-values for the relationship between dietary intake and radiation-induced survivorship syndromes among gynecological cancer-survivors and population controls.

DIETARY INTAKE	EXCESSIVE GAS DISCHARGE				EXCESSIVE MUCUS DISCHARGE				BLOOD DISCHARGE			
	CANCER SURVIVORS		POPULATION CONTROLS		CANCER SURVIVORS		POPULATION CONTROLS		CANCER SURVIVORS		POPULATION CONTROLS	
	Corr^a^	P^b^	Corr^a^	P^b^	Corr^a^	P^b^	Corr^a^	P^b^	Corr^a^	P^b^	Corr^a^	P^b^
Beans and lentils	-0.01	0.863	0.1	0.082	-0.08	0.064	-0.05	0.345	0.04	0.366	0.06	0.306
Cabbage and broccoli	0.01	0.748	0.06	0.271	-0.05	0.183	**-0.12**	**0.029**	0.02	0.627	0.06	0.254
Onion and garlic	0.00	0.912	0.04	0.444	0.03	0.445	0.02	0.754	0.03	0.472	0.03	0.551
Grated vegetables	0.07	0.112	0.02	0.737	-0.02	0.58	-0.02	0.693	0.13	0.002	0.03	0.549
Gluten	-0.03	0.529	0.05	0.378	**-0.08**	**0.049**	**0.11**	**0.044**	-0.03	0.415	-0.01	0.892
Citrus fruits	**0.1**	**0.018**	0.03	0.549	0.03	0.4	0.07	0.174	**0.18**	**<0.001**	0.01	0.793
Citrus fruits and vegetables	0.07	0.121	0.03	0.648	0	0.913	0.01	0.812	**0.17**	**<0.001**	0.02	0.701

^a^ Spearman correlation coefficient.

^b^ P-value

**Fig 1:**
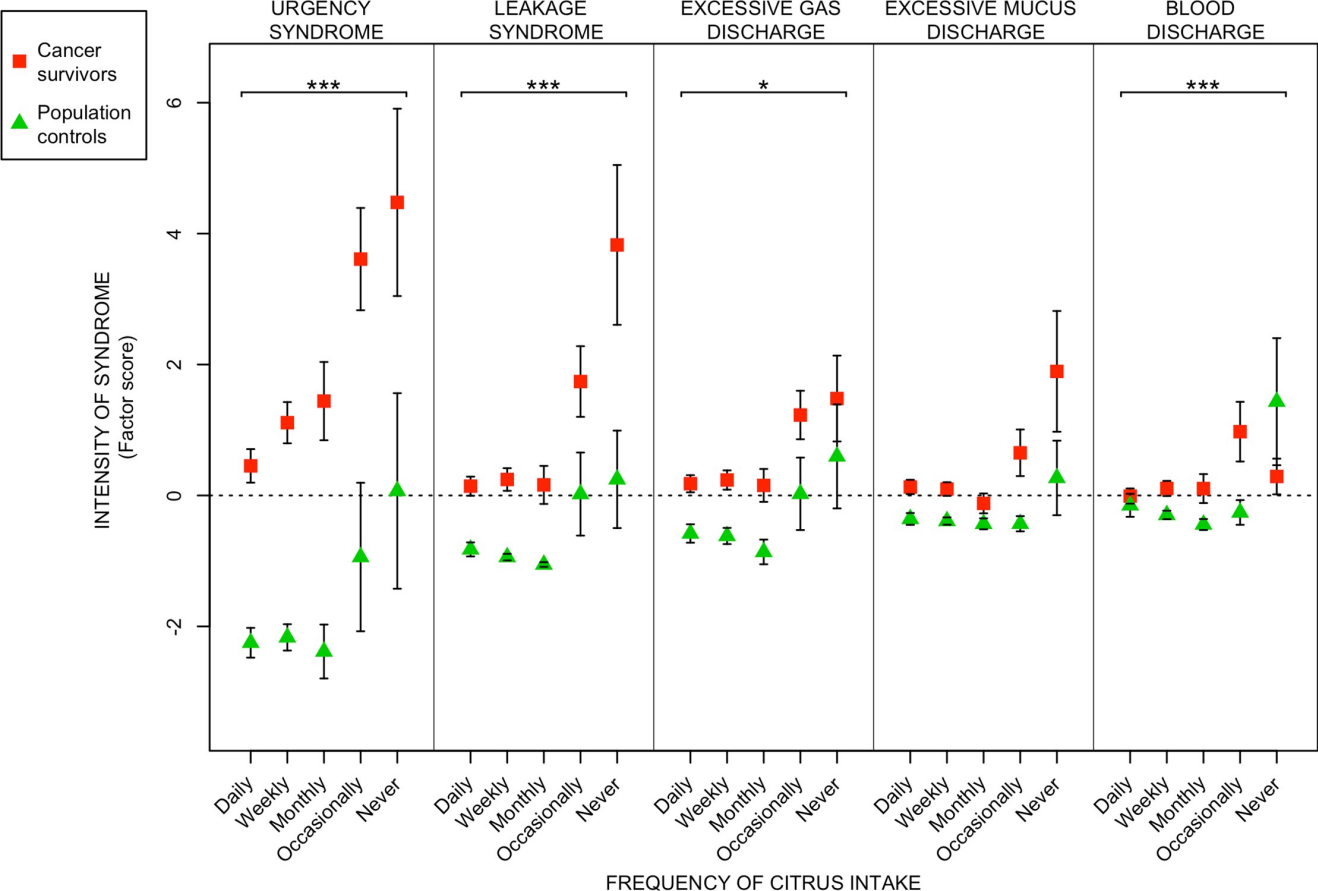
Relationships between radiation-induced survivorship syndromes and frequency of citrus intake. The figure depicts the relationships between frequency of citrus intake (x-axis) and intensities (factor scores) of the five radiation induced syndromes (y-axis: defecation-urgency syndrome, fecal-leakage syndrome, excessive gas discharge, excessive mucus discharge and blood discharge) among long-term gynecological cancer survivors and population controls. Red squares and green triangles denote the mean syndrome intensities within certain citrus intake frequency levels. Vertical black lines stretch between plus-minus the standard error of the mean. Asterisks correspond to significance levels of the Spearman correlations between syndrome intensities and frequency of citrus intake according to: p ≤ 0.001 - ***, 0.001 < p ≤ 0.01 - **. 0.01 < p ≤ 0.05 - *. Estimates of the Spearman correlations are found in Tables 2 and 3.

**Fig 2:**
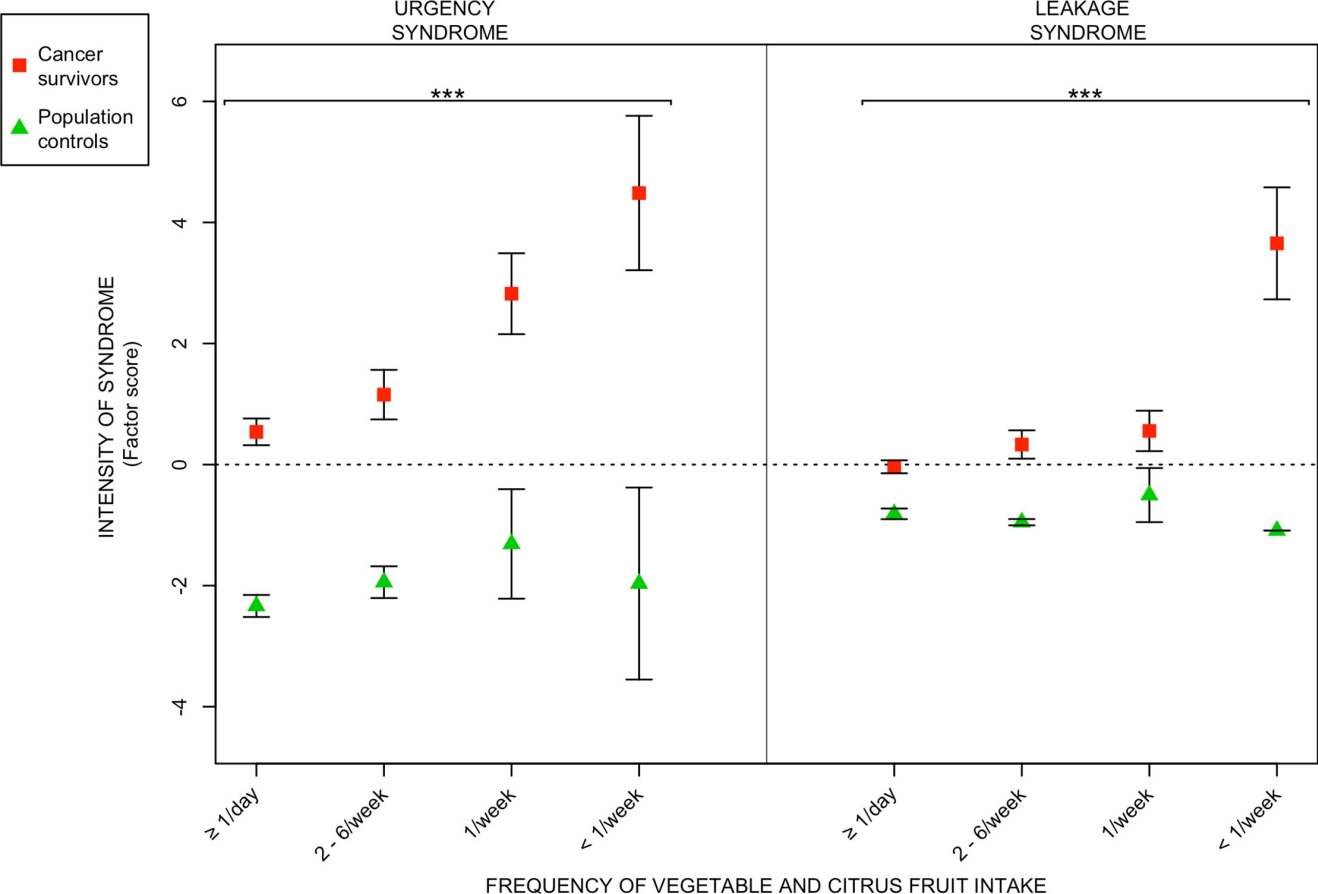
Relationship between radiation-induced survivorship syndromes and frequency of vegetable and citrus fruit intake. The figure depicts the relationships between frequency of combined intake of beans and lentils, cabbage and broccoli, onion and garlic, grated vegetables together with citrus fruits (x-axis) and intensities (factor scores) of the two radiation-induced survivorship syndromes (y-axis) defecation-urgency syndrome and fecal-leakage syndrome among long-term gynecological cancer survivors and population controls. Red squares and green triangles denote the mean syndrome intensities within certain vegetable and citrus intake frequency levels. Vertical black lines stretch between plus-minus the standard error of the mean. Asterisks correspond to significance levels of the Spearman correlations between syndrome intensities and frequency of vegetable and citrus intake according to: p ≤ 0.001 - ***, 0.001 < p ≤ 0.01 - **. 0.01 < p ≤ 0.05 - *. Estimates of the Spearman correlations are found in Tables 2 and 3.

**Fig 3:**
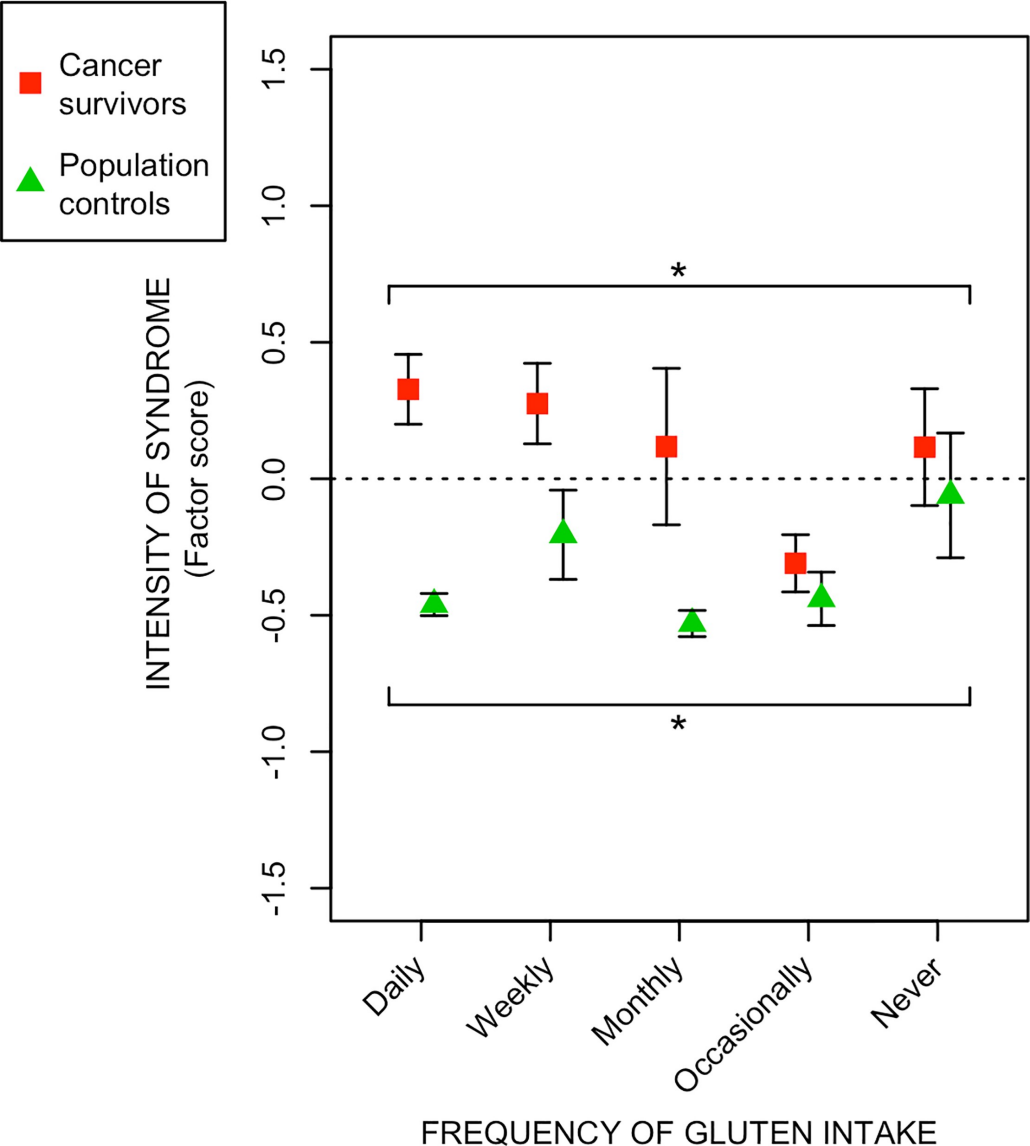
Relationship between intensity of excessive mucus discharge and frequency of gluten intake. The figure depicts the relationship between frequency of gluten intake (x-axis) and the intensity (factor scores) of the radiation induced syndrome (y-axis) excessive mucus discharge among long-term gynecological cancer survivors and population controls. Red squares/green triangles denote the mean syndrome intensity within certain gluten intake frequency levels. Vertical black lines stretch between plus-minus the standard error of the mean. Asterisks correspond to significance levels of the Spearman correlations between syndrome intensity and frequency of gluten intake according to: p ≤ 0.001 - ***, 0.001 < p ≤ 0.01 - **. 0.01 < p ≤ 0.05 - *. Estimates of the Spearman correlations are found in Tables 2 and 3.

In Figs 1 to 3, the relative measure of syndrome intensity (factor score; Y-axis), is plotted against increasing frequency of intake of the various food items; Fig 1) the intensity declined with increasing intake of citrus fruits, Fig 2) the intensity of defecation-urgency and fecal-leakage syndrome declined with increasing intake of vegetables and citrus fruits combined, Fig 3) for intake of gluten the trend was reversed, and the intensity of excessive mucus discharge was increased with increasing intake of gluten.

**Fig 4:**
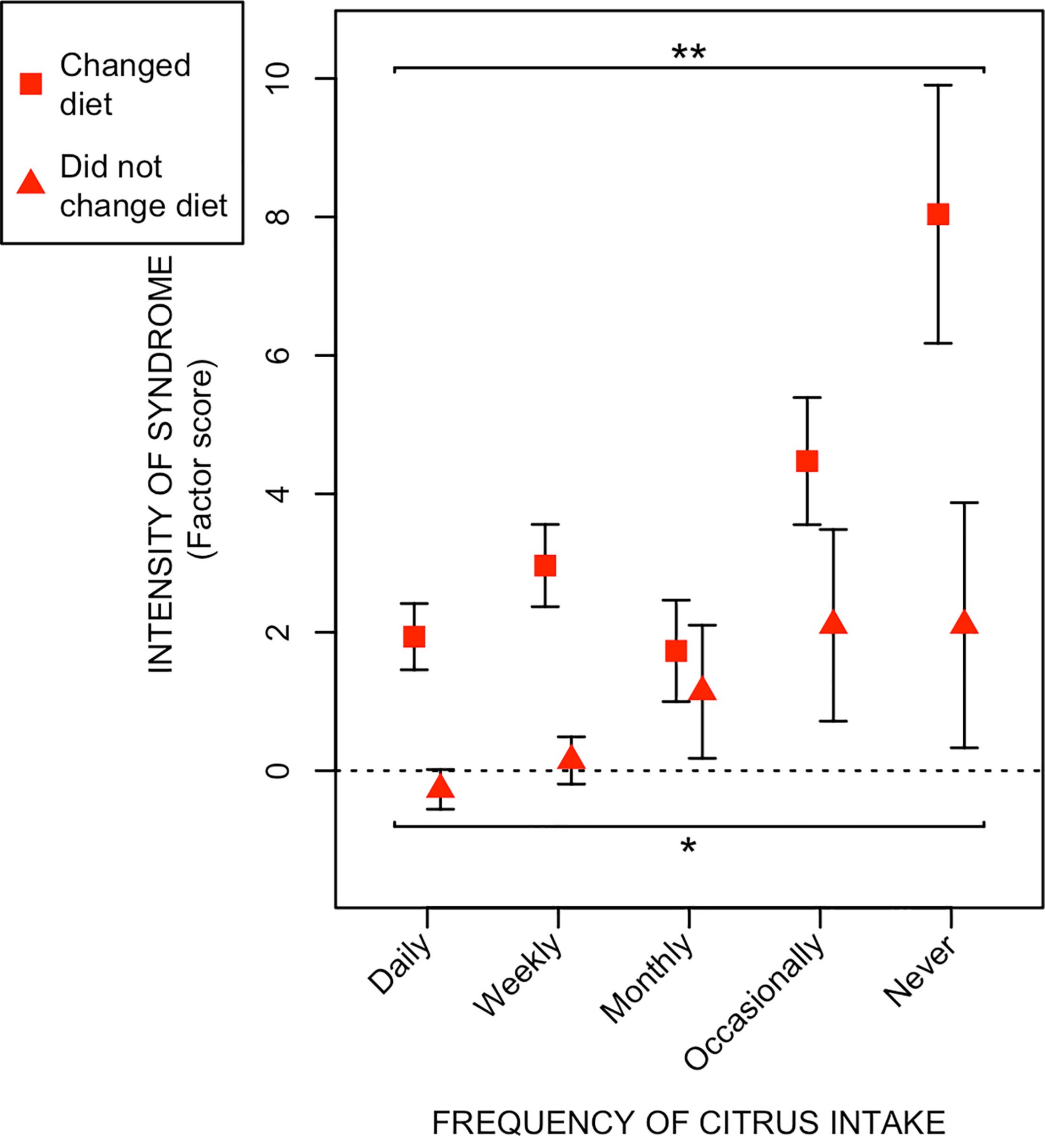
Relationship between radiation-induced defecation-urgency syndrome and frequency of citrus fruit intake, stratified by those who have changed and those who have not changed dietary behavior after radiotherapy. The figure depicts the relationship between frequency of citrus fruit intake (x-axis) and the intensity (factor scores) the defecation-urgency syndrome (y-axis) among long-term gynecological cancer survivors stratified according to having or not having made dietary changes after receiving radiotherapy (see the Methods section for detailed information). Red squares and green triangles denote the mean intensities of the syndromes within certain citrus intake frequency levels. Vertical black lines stretch between plus-minus the standard error of the mean. Asterisks correspond to significance levels of the Spearman correlations between syndrome intensity and frequency of citrus intake according to: p ≤ 0.001 - ***, 0.001 < p ≤ 0.01 - **. 0.01 < p ≤ 0.05 - *.

In Fig 4, the relationship between radiation-induced survivorship defecation-urgency syndrome and citrus fruit intake is stratified by those who have changed and those who have not changed their dietary behavior after radiotherapy. The intensity of defecation-urgency syndrome declined with increasing intake of citrus fruits in both groups, although the absolute intensity score was higher among those who had changed their dietary behavior after treatment.

Stratified analysis was performed to identify, if smoking, age or time since treatment confounded the relationship between the radiation-induced defecation-urgency or fecal-leakage syndrome and citrus fruit intake. The analysis was stratified by those who were non-smokers and those who were former or current smokers, or time since treatment less than median (6.3 year) and above, or age less than median (65.1) and above. The results did not differ substantially between the strata for any of the analyses (data not shown).

## Discussion

This is the first study evaluating the relationship between dietary intake and five different radiation-induced longstanding syndromes. Based on information from 623 women, three to fifteen years after radiotherapy, we found that frequent intake of citrus fruits and vegetables decreased the intensity of defecation-urgency syndrome, fecal-leakage syndrome, excessive gas discharge and blood discharge. The strongest relationship was seen for the defecation-urgency syndrome. In contrast, the intensity of excessive mucus discharge was increased with increasing intake of gluten.

Our results are supported by experimental data that show that compounds in fruits and vegetables such as soluble fiber protect against radiation-induced damage of the gut. However, the data from clinical studies are sparse [8, 9, 24–28]. Non-starch complex polysaccharides, found in high amounts in plant cell walls, seem to have several different functions that improve the colonic function (listed in Table 4).

**Table 4:** Components in the diet and their possible pathophysiological processes causing the effect on radiation-induced survivorship symptoms.

Components in the diet	Possible pathophysiological processes causing the effect.
Non-starch polysaccharides e.g. pectin	I. Oral intake increased levels of tissue hydroxyproline and decreased MMP-2 in colon in mice^[^^29^^]^. II. Increases transit rate which in turn lowers colonic pH ^[^^30^^]^. III. Increases intestinal stem cell genes expression^[^^12^^]^ IV. Increases intestinal crypt survival after ionizing radiation^[^^12^^]^ V. Act as prebiotics^[^^14^^]^ VI. Gelling effect / mechanic effect—prevent loose stools^[^^31^^]^ VII. Decreases inflammation processes^[^^32^^]^ VIII. Delays gastric emptying^[^^33^^]^ VIIII. Binds bile acids in the small intestine^[^^31^^,^ ^34^^]^ X. Hinders colonic mucus barrier erosion, by acting as substrate for microbiota^[^^10^^] [^^17^^]^
Antioxidants, flavonoids, phytochemicals, indols	I. Decreases inflammation processes^[^^35^^]^ II. Restores intestinal barrier^[^^35^^]^
Short-chain fatty acids (SCFA), butyrate	I. Intestinal barrier regulation^[^^16^^]^ II. Antidiarrheal function ^[^^16^^]^ III. Anti-inflammatory ^[^^16^^]^ IV. Anti-fibrogenic effect ^[^^13^^,^ ^15^^]^

In humans, dietary soluble fiber, like pectin, are not digested by endogenous enzymes in the small intestine but are degraded by the colon microbiota. The soluble fiber has a gelling effect in the digestive tract, slowing down digestion. By acting as prebiotics, the polysaccharides are fermented into short chain fatty acids in the large intestine [14]. A diet rich in non-starch polysaccharides is also believed to increase the transit rate through the colon, which in turn lowers the colonic pH. A lower pH 5.5 ameliorates production of short–chain fatty acids, in particular butyrate [30]. Short–chain fatty acids, especially butyrate, provide the colonic epithelial cells with up to 70% of their energy requirements [17]. Butyrate is suggested to have a function in the regulation of the gut wall barrier, an anti-inflammatory, anti-diarrheal and anti-fibrinogenic function, as well as preventing mucosal atrophy [15, 16, 36]. Pectin is suggested to improve colonic function [31] and has anti-inflammatory properties [32, 35]. In an experimental study on mice, pectin increased intestinal crypt stem cell survival after radiation [12]. An oral intake of dietary fiber increased levels of hydroxyproline, a component in collagen, in rats given radiotherapy [29]. A modified form of pectin has been found to increase the ^137^Cs clearance, a radio-isotope produced during uranium fission. When eaten for several days, toxic elements seem to be chelated by the pectin and then eliminated in the urine [11]. In a recent mouse experiment, dietary apple pectin ameliorated the radiation-induced increase in submucosa thickness and protected the terminal ileum against radiation-induced fibrosis [13]. In addition, the irradiated mice had a markedly decreased diversity of intestinal microbiota, while the pectin-treated mice had a smaller decrease in diversity. The pectin-treated mice also had markedly higher concentrations of short–chain fatty acids in their feces. However, extrapolating knowledge gained from animal models should be done with caution, since species differences, and even strain differences affect the response to radiation. In addition, the irradiation procedure in animal models is, for practical reasons, a simplification of pelvic radiotherapy. Nevertheless, the hall-marks of radiation-induced injury to the human intestines such as inflammation of the intestinal wall, an increase in serum cytokines, stem cell loss, crypt degeneration, angiogenesis and fibrosis are readily reproduced in animal models. By focusing on these similarities, animal models have provided the foundation of today’s understanding of how we react to radiation exposure, and how various external factors can modify this response [37].

In contrast to the beneficial effects of citrus fruits and vegetables on the defecation-urgency syndrome, increased intake of gluten promoted excessive mucus discharge. This may be a result of radiation-induced damage making the intestine more vulnerable, triggering a non-celiac gluten sensitivity. It can also be due to an interaction from, or confounding effect of, something else in the diet, correlated to gluten intake [38, 39]. The sensitivity to gluten may also be a result of an underlying genetic tendency towards coeliac disease, triggered by the radiation-induced immunosuppression [40]. However, we did not find any effect of gluten intake on defecation-urgency syndrome, a syndrome for which the symptoms are similar to those of coeliac disease. This suggests that the gluten sensitivity presented here is not related to coeliac disease.

The normal function of the gut is highly dependent on the type, diversity and the number of microbiota, which in turn depends partly on dietary habits [41, 42]. The concentration of soluble or insoluble fiber in the diet affects the composition and function of the gut microbiota [18, 43]. The colonic mucus barrier serves as a primary defense against pathogens and toxic compounds [10]. A diet low in fiber leads to starvation of the gut microbiota, which results in starting use of the host-secreted mucus glycoproteins as a nutrient source. This leads in turn to erosion of the colonic mucus barrier [10]. The erosion may facilitate migration of pro-inflammatory species such as *Bacteroides fragilis* through the gut wall [44].

Previously, we have identify that the symptoms exist in the background population [19], caused by other factors than radiation. At a certain intensity of symptoms, we can suspect chronic pathophysiological processes generated by the radiation therapy. Radiation causes degeneration of crypts in the gut wall and over time the gut wall may turn into rigid, dysfunctional fibrotic tissue. Crypts probably degenerate when the last stem cell has been depleted or made non-functional by lack of supportive cells [45, 46]. The damaged gut wall may regain normal function by recruiting non-dividing radio-resistant “reserve” stem cells that divide and repopulate the radiosensitive stem-cell population and new crypts can be formed by crypt fission [46, 47]. Probably, inflammation in the gut wall hinders intestinal restoration and results in fibrosis, and the inflammation may occur when the inner mucus layer fails to protect the gut wall from being exposed to pro-inflammatory bacteria [10]. Moreover, bacterial invasion may be enhanced when the epithelial tight junctions are injured by radiation [45, 48–50]. Such a mechanism could turn into a vicious cycle, self-propelling further inflammation, ischemia, gut wall leakage and ultimately degeneration of crypts and fibrosis of the gut wall [5, 45, 51]. Dietary components or probiotics may hinder or dampen this inflammation by two mechanisms: one by keeping the two mucus layers intact, the other by hindering a gut-wall starvation that makes the wall vulnerable to inflammatory agents [52].

Of the few randomized clinical studies investigating possible effects of soluble fiber on radiation-induced symptoms, both low and high fiber intake have been studied. In a recent study, 166 patients undergoing pelvic radiotherapy were randomly assigned to receive low-fiber, habitual-fiber or high-fiber dietary advice and treatment. The high-fiber diet resulted in reduced gastrointestinal toxicity both acutely and at 1 y compared with habitual-fiber intake [28]. The effect of fiber supplementation with Metamucil (Psyllium) on radiation-induced diarrhea was determined in a randomized trial of 60 patients. Metamucil was shown to significantly decrease the incidence and severity of diarrhea [8]. In another randomized trial [26], radiotherapy-induced gastrointestinal symptoms were compared between prostate cancer patients who were either advised to reduce insoluble dietary fiber and lactose, or to continue to eat their normal diet. The results showed that the dietary intervention was not superior to a normal diet in preventing the symptoms. In some cases, the symptoms, for example stool leakage and blood in stools, were worse among those in the intervention group than among those in the control group. Interestingly, the control group ate more high-fiber vegetables than the intervention group. If vegetables have a protective effect, this may explain the results of the study. In another study, patients with irritable bowel syndrome were given pectin powder daily or placebo in a six-weeks randomized trial. The pectin group experienced a greater reduction in composite symptom scores and Bristol stool scale scores, and pectin acted as a prebiotic and significantly enhanced fecal bifidobacteria and decreased total *Clostridium sp*. [27]. A just published Cochrane-review found a low-certainty evidence, suggesting that a high-fiber diet may lead to better intestinal health one year after radiation treatment [7]. The results were mainly based on Wedlake et al. which also used the highest fiber intake intervention, targeting of >18 g non-starch polysaccharide per day.

Today, there exists no consensus on dietary recommendations before or after radiotherapy, due to the lack of evidence. In Sweden, advice from clinical practitioners is, in general, given only to those patients who ask for it. Examples of such advice are to decrease the intake of lactose, fiber, cereals, or gas-producing food, take a prescription medication like Dimetikon and Loperamid, and if the patient is undernourished increase fat, protein and water intake. In descriptive studies where patients have been interviewed about the dietary practice they use to manage their gastrointestinal symptoms, some reported that elimination of brans, raw vegetables, brassica vegetables, or beans helped, whereas others reported that such interventions did not help [53, 54]. Vegetables like those in the genus brassica and beans or raw vegetables can cause in some individuals gastrointestinal symptoms and discomfort, even if there is no radiation-induced gastrointestinal damage, often due to increased gas formation [55]. Therefore, dietary advice to patients should be given on an individual level, recommending a diet high in soluble fiber if tolerated by the patient.

Strengths of our study include its large size, the population-based design, and the use of a novel metrics for the intensity of the specific syndromes reflecting specific radiation-induced survivorship diseases. Using these metrics instead of single symptoms or a score that nonspecifically reflects different radiation-induced survivorship diseases with varying pathophysiology, we will remove noise that otherwise would have diluted the effect measures. The ethnic homogeneity of our study population (only 8% of the women were born outside Northern Europe) reduces the risk of confounding by unmeasured factors, although it may limit the generalisability of the results to other populations or to men. We cannot exclude that the associations are different among survivors who did not participate. Since information on dietary intake was only collected once and after radiation, reversed causality could be argued. Even if information on symptoms and diet was collected at the same time, symptoms in the past could have changed the dietary behavior among the patients. However, the intensity of syndrome declined with increasing intake of citrus fruits both for those who had, or had not, changed their dietary behaviour after radiotherapy. In addition, if patients with a high burden of symptom had changed their diet, our results concerning gluten should have been similar to results for citrus fruits and vegetables. In contrast, those with a daily intake of gluten reported more often symptoms than those with no intake of gluten. There is a popular belief that gluten can increase problems from the gut and intestine. However, the same is not true for intake of fruit and vegetables; therefore, it is more likely that symptomatic patients will stop eating gluten than fruits and vegetables. In addition, the cancer survivors included in this study were treated between 1991 and 2003, when general dietary recommendations given by clinicians were few. Recommendations given did not include a recommendation to stop eating fruit and vegetables (interview with clinicians, unpublished data). Moreover, dietary habits are often life-long. Thus, we have reason to believe that the dietary behavior presented in this study most likely reflects dietary habits that were established well before radiotherapy was started.

One limitation is that we do not have information on total dietary intake. As a result, misclassification of fruit and vegetable intake may exist; even so, the misclassification is probably non-differential (the same question was answered by all participants regardless of symptom burden), which may lead to an attenuation of the risk estimate. The lack of total dietary intake measurement makes us unable to perform statistical adjustment for potential dietary confounders. However, the most likely confounding dietary factors such as intake of dairy products, spicy food or fatty food were estimated, and none of them was statistically significantly correlated to the intensity of the various radiation-induced survivorship diseases. Concerning smoking, age and time since treatment we found a small, if any, confounding effect concerning the relation between citrus and vegetables and intensity of the urgency syndrome. However, we cannot exclude residual confounding of unmeasured or unknown risk factors. With regard to the study design, our results needs to be confirmed in a clinical dietary intervention trial.

In conclusion, our results indicate that high intake of citrus fruits and vegetables lowers the intensity of radiation-induced cancer survivorship diseases. Evidence from experimental data clearly supports our findings. Our preliminary experiments in a mouse model, specifically developed to study the presence of degenerating crypts and fibrosis in distal colon and rectum after radiotherapy [56], suggest that different levels and type of dietary fiber affect the severity of radiation-damage in mouse colon. Therefore, it may be fruitful to identify individual dietary fiber, or groups of fiber, that could reduce the occurrence of radiation-induced intestinal damage. In future clinical trials, manipulation of diet before, during and after radiotherapy could provide us with new, relatively easy and affordable means to prevent or reduce radiation-induced survivorship diseases of the gut. A special emphasis should be placed on preventing the defecation-urgency syndrome, which is reportedly the most debilitating syndrome for the cancer survivor [19]. When it comes to optimizing dietary interventions with specific individual dietary fiber or groups of fiber, using criteria such as water solubility, molecular composition or other, more data from observational and pre-clinical studies are needed before clinical trials can be considered ethically defensible.

## Supporting information

S1 File Survey questions. Questions regarding symptoms related to intestinal health, presented in original language and translation to English.
(PDF)

S1 Dataset Dataset of population controls. (TXT)

S2 Dataset Dataset of cancer survivors. (TXT)
