# Supplementary material for: Intake of citrus fruits and vegetables and the intensity of defecation urgency syndrome among gynecological cancer survivors
Source: PLoS One. 2019 Jan 2;14(1):e0208115. doi: 10.1371/journal.pone.0208115 (PMC6314594; doi:10.1371/journal.pone.0208115)
Supplement: S1 File — Questions regarding symptoms related to intestinal health, presented in original language and translation to English. (PDF) [file pone.0208115.s001.pdf]

# S1 File

# Survey questions

## Swedish

1. Har Du haft lös avföring, det senaste halvåret?

   - ❑ Nej
   - ❑ Ja, vid enstaka tillfällen
   - ❑ Ja, åtminstone någon gång varje månad
   - ❑ Ja, åtminstone någon gång varje vecka
   - ❑ Ja, åtminstone tre gånger varje vecka
   - ❑ Ja, åtminstone någon gång varje dag

2. Har Du haft hård avföring som har varit svår att få ut, det senaste halvåret?

   - ❑ Nej
   - ❑ Ja, vid enstaka tillfällen
   - ❑ Ja, åtminstone någon gång varje månad
   - ❑ Ja, åtminstone någon gång varje vecka
   - ❑ Ja, åtminstone tre gånger varje vecka
   - ❑ Ja, åtminstone någon gång varje dag

3. Hur har Din förmåga varit att vid behov kunna krysta ut avföringen, det senaste halvåret?

   - ❑ ***Inte aktuellt,*** jag har inte haft behov av att krysta ut avföringen, det senaste halvåret

   - ❑ Ingen förmåga
   - ❑ Liten förmåga
   - ❑ Måttlig förmåga
   - ❑ Stor förmåga

4. Har Du haft en känsla av att inte ha tömt tarmen fullständigt, det senaste halvåret?

   - ❑ Nej
   - ❑ Ja, vid enstaka tillfällen
   - ❑ Ja, åtminstone någon gång varje månad
   - ❑ Ja, åtminstone någon gång varje vecka
   - ❑ Ja, åtminstone tre gånger varje vecka
   - ❑ Ja, åtminstone någon gång varje dag

5. Har Du återvänt till toaletten inom en timme efter avföring för att återigen tömma tarmen, det senaste halvåret?

   - ❑ Nej
   - ❑ Ja, vid enstaka tillfällen
   - ❑ Ja, åtminstone någon gång varje månad

- ❑ Ja, åtminstone någon gång varje vecka
- ❑ Ja, åtminstone tre gånger varje vecka
- ❑ Ja, åtminstone någon gång varje dag

6. Har Du haft känsla av uppblåsthet i magen, det senaste halvåret?

- ❑ Nej
- ❑ Ja, vid enstaka tillfällen
- ❑ Ja, åtminstone någon gång varje månad
- ❑ Ja, åtminstone någon gång varje vecka
- ❑ Ja, åtminstone tre gånger varje vecka
- ❑ Ja, åtminstone någon gång varje dag

7. Har det kommit blod från tarmen, det senaste halvåret?

- ❑ Nej
- ❑ Ja, vid enstaka tillfällen
- ❑ Ja, åtminstone någon gång varje månad
- ❑ Ja, åtminstone någon gång varje vecka
- ❑ Ja, åtminstone tre gånger varje vecka
- ❑ Ja, åtminstone någon gång varje dag

8. Har det kommit slem från tarmen, det senaste halvåret?

- ❑ Nej
- ❑ Ja, vid enstaka tillfällen
- ❑ Ja, åtminstone någon gång varje månad
- ❑ Ja, åtminstone någon gång varje vecka
- ❑ Ja, åtminstone tre gånger varje vecka
- ❑ Ja, åtminstone någon gång varje dag

9. Har Du haft klåda vid ändtarmsöppningen, det senaste halvåret?

- ❑ Nej
- ❑ Ja, vid enstaka tillfällen
- ❑ Ja, åtminstone någon gång varje månad
- ❑ Ja, åtminstone någon gång varje vecka
- ❑ Ja, åtminstone tre gånger varje vecka
- ❑ Ja, åtminstone någon gång varje dag

10. Har Du haft smärta i ändtarmsöppningen, det senaste halvåret?

- ❑ Nej
- ❑ Ja, vid enstaka tillfällen
- ❑ Ja, åtminstone någon gång varje månad
- ❑ Ja, åtminstone någon gång varje vecka

- ❑ Ja, åtminstone tre gånger varje vecka
- ❑ Ja, åtminstone någon gång varje dag

11. Har Du haft plötsligt påkomna avföringsträngningar som krävt omedelbart toalettbesök, det senaste halvåret?

- ❑ Nej
- ❑ Ja, vid enstaka tillfällen
- ❑ Ja, åtminstone någon gång varje månad
- ❑ Ja, åtminstone någon gång varje vecka
- ❑ Ja, åtminstone tre gånger varje vecka
- ❑ Ja, åtminstone någon gång varje dag

12. Hur länge har Du kunnat hålla avföringen vid trängningar, det senaste halvåret?

- ❑ ***Inte aktuellt,*** jag har inte haft avföringsträngningar, det senaste halvåret

- ❑ Kortare tid än 1 minut
- ❑ Mellan 1 och 5 minuter
- ❑ Mellan 5 och 10 minuter
- ❑ Mellan 10 och 30 minuter
- ❑ 30 minuter eller längre

13. Har Du haft <u>omedelbart</u> behov av en toalett om Du behövt tömma tarmen, det senaste halvåret?

- ❑ Nej
- ❑ Ja, vid enstaka tillfällen
- ❑ Ja, åtminstone någon gång varje månad
- ❑ Ja, åtminstone någon gång varje vecka
- ❑ Ja, åtminstone tre gånger varje vecka
- ❑ Ja, åtminstone någon gång varje dag

14. Har Du utan förvarning läckt avföring i kläderna, trots att Du tidigare tömt tarmen, det senaste halvåret?

- ❑ Nej
- ❑ Ja, vid enstaka tillfällen
- ❑ Ja, åtminstone någon gång varje månad
- ❑ Ja, åtminstone någon gång varje vecka
- ❑ Ja, åtminstone tre gånger varje vecka
- ❑ Ja, åtminstone någon gång varje dag

15. Har Du utan förvarning tömt <u>all</u> avföring i kläderna, det senaste halvåret?

- ❑ Nej
- ❑ Ja, vid enstaka tillfällen
- ❑ Ja, åtminstone någon gång varje månad
- ❑ Ja, åtminstone någon gång varje vecka
- ❑ Ja, åtminstone tre gånger varje vecka

- ❑ Ja, åtminstone någon gång varje dag

16. Har det hänt att Du inte har kunnat hålla kvar gas (fjärtar, fisar) i ändtarmen när det behövts, det senaste halvåret?

- ❑ Nej
- ❑ Ja, vid enstaka tillfällen
- ❑ Ja, åtminstone någon gång varje månad
- ❑ Ja, åtminstone någon gång varje vecka
- ❑ Ja, åtminstone tre gånger varje vecka
- ❑ Ja, åtminstone någon gång varje dag

17. Har Du haft ljudliga gasavgångar (fjärtar, fisar) som Du inte kunnat stoppa, det senaste halvåret?

- ❑ Nej
- ❑ Ja, vid enstaka tillfällen
- ❑ Ja, åtminstone någon gång varje månad
- ❑ Ja, åtminstone någon gång varje vecka
- ❑ Ja, åtminstone tre gånger varje vecka
- ❑ Ja, åtminstone någon gång varje dag

18. Har Du haft illaluktande gasavgångar (fjärtar, fisar) som Du inte kunnat stoppa, det senaste halvåret?

- ❑ Nej
- ❑ Ja, vid enstaka tillfällen
- ❑ Ja, åtminstone någon gång varje månad
- ❑ Ja, åtminstone någon gång varje vecka
- ❑ Ja, åtminstone tre gånger varje vecka
- ❑ Ja, åtminstone någon gång varje dag

19. Har Du haft läckage av slem från ändtarmen när Du varit <u>vaken</u>, det senaste halvåret?

- ❑ Nej
- ❑ Ja, vid enstaka tillfällen
- ❑ Ja, åtminstone någon gång varje månad
- ❑ Ja, åtminstone någon gång varje vecka
- ❑ Ja, åtminstone tre gånger varje vecka
- ❑ Ja, åtminstone någon gång varje dag

20. Har Du haft läckage av blod från ändtarmen när Du varit <u>vaken,</u> det senaste halvåret?

- ❑ Nej
- ❑ Ja, vid enstaka tillfällen
- ❑ Ja, åtminstone någon gång varje månad
- ❑ Ja, åtminstone någon gång varje vecka

- ❑ Ja, åtminstone tre gånger varje vecka
- ❑ Ja, åtminstone någon gång varje dag

21. Har Du haft läckage av lös avföring när Du varit vaken, det senaste halvåret?

- ❑ Nej
- ❑ Ja, vid enstaka tillfällen
- ❑ Ja, åtminstone någon gång varje månad
- ❑ Ja, åtminstone någon gång varje vecka
- ❑ Ja, åtminstone tre gånger varje vecka
- ❑ Ja, åtminstone någon gång varje dag

22. Har Du haft läckage av fast avföring när Du varit vaken, det senaste halvåret?

- ❑ Nej
- ❑ Ja, vid enstaka tillfällen
- ❑ Ja, åtminstone någon gång varje månad
- ❑ Ja, åtminstone någon gång varje vecka
- ❑ Ja, åtminstone tre gånger varje vecka
- ❑ Ja, åtminstone någon gång varje dag

23. Har Du haft läckage av slem från ändtarmen, när Du sovit, det senaste halvåret?

- ❑ Nej
- ❑ Ja, vid enstaka tillfällen
- ❑ Ja, åtminstone någon gång varje månad
- ❑ Ja, åtminstone någon gång varje vecka
- ❑ Ja, åtminstone tre gånger varje vecka
- ❑ Ja, åtminstone någon gång varje dag

24. Har Du haft läckage av blod från ändtarmen, när Du sovit, det senaste halvåret?

- ❑ Nej
- ❑ Ja, vid enstaka tillfällen
- ❑ Ja, åtminstone någon gång varje månad
- ❑ Ja, åtminstone någon gång varje vecka
- ❑ Ja, åtminstone tre gånger varje vecka
- ❑ Ja, åtminstone någon gång varje dag

25. Har Du haft läckage av lös avföring, när Du sovit, det senaste halvåret?

- ❑ Nej
- ❑ Ja, vid enstaka tillfällen
- ❑ Ja, åtminstone någon gång varje månad
- ❑ Ja, åtminstone någon gång varje vecka

- ❑ Ja, åtminstone tre gånger varje vecka
- ❑ Ja, åtminstone någon gång varje dag

26. Har Du haft läckage av fast avföring när Du sovit, det senaste halvåret?

- ❑ Nej
- ❑ Ja, vid enstaka tillfällen
- ❑ Ja, åtminstone någon gång varje månad
- ❑ Ja, åtminstone någon gång varje vecka
- ❑ Ja, åtminstone tre gånger varje vecka
- ❑ Ja, åtminstone någon gång varje dag

27. Har Du tyckt att Du har luktat avföring någon gång, det senaste halvåret?

- ❑ Nej
- ❑ Ja, vid enstaka tillfällen
- ❑ Ja, åtminstone någon gång varje månad
- ❑ Ja, åtminstone någon gång varje vecka
- ❑ Ja, åtminstone tre gånger varje vecka
- ❑ Ja, åtminstone någon gång varje dag

28. Har Du haft smärtor i buken, det senaste halvåret?

- ❑ Nej
- ❑ Ja, vid enstaka tillfällen
- ❑ Ja, åtminstone någon gång varje månad
- ❑ Ja, åtminstone någon gång varje vecka
- ❑ Ja, åtminstone tre gånger varje vecka
- ❑ Ja, åtminstone någon gång varje dag

# English

1. Have you had a loose bowel movement during the past half year?

- ❑ No
- ❑ Yes, on occasion
- ❑ Yes, at least once a month
- ❑ Yes, at least once a week
- ❑ Yes, at least three times a week
- ❑ Yes, at least once a day

2. Have you had a hard bowel movement that has been hard to get out during the past half year?

- ❑ No
- ❑ Yes, on occasion
- ❑ Yes, at least once a month
- ❑ Yes, at least once a week

- ❑ Yes, at least three times a week
- ❑ Yes, at least once a day

3. Have you been able to push out stools when you needed to during the past half year?

- ❑ ***Not a problem,*** I have not needed to push hard to have a bowel movement during the past half year

- ❑ Not able
- ❑ Slightly able
- ❑ Moderately able
- ❑ Completely able

4. Do you have the feeling that you have not been able to completely empty your bowels during the past half year?

- ❑ No
- ❑ Yes, on occasion
- ❑ Yes, at least once a month
- ❑ Yes, at least once a week
- ❑ Yes, at least three times a week
- ❑ Yes, at least once a day

5. Have you had to go to the toilet within an hour after a bowel movement to once again empty your bowels during the past half year?

- ❑ No
- ❑ Yes, on occasion
- ❑ Yes, at least once a month
- ❑ Yes, at least once a week
- ❑ Yes, at least three times a week
- ❑ Yes, at least once a day

6. Have you had the feeling that your stomach is bloated during the past half year?

- ❑ No
- ❑ Yes, on occasion
- ❑ Yes, at least once a month
- ❑ Yes, at least once a week
- ❑ Yes, at least three times a week
- ❑ Yes, at least once a day

7. Has blood come out from your bowels during the past half year?

- ❑ No
- ❑ Yes, on occasion
- ❑ Yes, at least once a month
- ❑ Yes, at least once a week
- ❑ Yes, at least three times a week
- ❑ Yes, at least once a day

8. Has mucus come out from your bowels during the past half year?

- ❑ No
- ❑ Yes, on occasion
- ❑ Yes, at least once a month
- ❑ Yes, at least once a week
- ❑ Yes, at least three times a week
- ❑ Yes, at least once a day

9. Have you had itching in your anus during the past half year?

- ❑ No
- ❑ Yes, on occasion
- ❑ Yes, at least once a month
- ❑ Yes, at least once a week
- ❑ Yes, at least three times a week
- ❑ Yes, at least once a day

10. Have you had pain in your anus during the past half year?

- ❑ No
- ❑ Yes, on occasion
- ❑ Yes, at least once a month
- ❑ Yes, at least once a week
- ❑ Yes, at least three times a week
- ❑ Yes, at least once a day

11. Have you had a sudden need to defecate that has required an immediate visit to the toilet during the past half year?

- ❑ No
- ❑ Yes, on occasion
- ❑ Yes, at least once a month
- ❑ Yes, at least once a week
- ❑ Yes, at least three times a week
- ❑ Yes, at least once a day

12. How long have you been able to keep from defecating when you have had an urgent need to defecate during the past half year?

❑ ***Not at present,*** I have not had the need to defecate during the past half year.

❑ Less than 1 minute
❑ Between 1 and 5 minutes
❑ Between 5 and 10 minutes
❑ Between 10 and 30 minutes
❑ 30 minutes or longer

13. Have you had an immediate need for a toilet if you needed to defecate during the past half year?

❑ No
❑ Yes, on occasion
❑ Yes, at least once a month
❑ Yes, at least once a week
❑ Yes, at least three times a week
❑ Yes, at least once a day

14. Have you without forewarning leaked feces into your clothing even though you had earlier emptied your bowels during the past half year?

❑ No
❑ Yes, on occasion
❑ Yes, at least once a month
❑ Yes, at least once a week
❑ Yes, at least three times a week
❑ Yes, at least once a day
❑

15. Have you without forewarning completely emptied your bowels into your clothing during the past half year?

❑ No
❑ Yes, on occasion
❑ Yes, at least once a month
❑ Yes, at least once a week
❑ Yes, at least three times a week
❑ Yes, at least once a day

16. Has it happened that you have not been able to keep from releasing gas (farting) from your bowels when needed during the past half year?

❑ No
❑ Yes, on occasion
❑ Yes, at least once a month
❑ Yes, at least once a week
❑ Yes, at least three times a week

- ❑ Yes, at least once a day

17. Have you had audible release of gas (farts) that you could not prevent during the past half year?

- ❑ No
- ❑ Yes, on occasion
- ❑ Yes, at least once a month
- ❑ Yes, at least once a week
- ❑ Yes, at least three times a week
- ❑ Yes, at least once a day

18. Have you had smelly release of gas (farts) that you could not stop during the past half year?

- ❑ No
- ❑ Yes, on occasion
- ❑ Yes, at least once a month
- ❑ Yes, at least once a week
- ❑ Yes, at least three times a week
- ❑ Yes, at least once a day

19. Have you had leakage of mucus from your rectum when you have been awake during the past half year?

- ❑ No
- ❑ Yes, on occasion
- ❑ Yes, at least once a month
- ❑ Yes, at least once a week
- ❑ Yes, at least three times a week
- ❑ Yes, at least once a day

20. Have you had leakage of blood from your rectum when you were awake during the past half year?

- ❑ No
- ❑ Yes, on occasion
- ❑ Yes, at least once a month
- ❑ Yes, at least once a week
- ❑ Yes, at least three times a week
- ❑ Yes, at least once a day

21. Have you had leakage of loose stools when you have been awake during the past half year??

- ❑ No
- ❑ Yes, on occasion

- ❑ Yes, at least once a month
- ❑ Yes, at least once a week
- ❑ Yes, at least three times a week
- ❑ Yes, at least once a day

22. Have you had leakage of solid stools when you have been awake during the past half year?

- ❑ No
- ❑ Yes, on occasion
- ❑ Yes, at least once a month
- ❑ Yes, at least once a week
- ❑ Yes, at least three times a week
- ❑ Yes, at least once a day

23. Have you had leakage of mucus from your rectum when you were sleeping during the past half year?

- ❑ No
- ❑ Yes, on occasion
- ❑ Yes, at least once a month
- ❑ Yes, at least once a week
- ❑ Yes, at least three times a week
- ❑ Yes, at least once a day

24. Have you had leakage of blood from your rectum when you were sleeping during the past half year?

- ❑ No
- ❑ Yes, on occasion
- ❑ Yes, at least once a month
- ❑ Yes, at least once a week
- ❑ Yes, at least three times a week
- ❑ Yes, at least once a day

25. Have you had leakage of loose stools when you were sleeping during the past half year?

- ❑ No
- ❑ Yes, on occasion
- ❑ Yes, at least once a month
- ❑ Yes, at least once a week
- ❑ Yes, at least three times a week
- ❑ Yes, at least once a day

26. Have you had leakage of solid stools when you were sleeping during the past half year?

- ❑ No
- ❑ Yes, on occasion
- ❑ Yes, at least once a month
- ❑ Yes, at least once a week
- ❑ Yes, at least three times a week
- ❑ Yes, at least once a day

27. Have you felt that you have smelled your stools at some time during the past half year?

- ❑ No
- ❑ Yes, on occasion
- ❑ Yes, at least once a month
- ❑ Yes, at least once a week
- ❑ Yes, at least three times a week
- ❑ Yes, at least once a day

28. Have you had pain in your abdomen during the past half year?

- ❑ No
- ❑ Yes, on occasion
- ❑ Yes, at least once a month
- ❑ Yes, at least once a week
- ❑ Yes, at least three times a week
- ❑ Yes, at least once a day
